# Re-positive Cases of Nucleic Acid Tests in Discharged Patients With COVID-19: A Follow-Up Study

**DOI:** 10.3389/fmed.2020.00349

**Published:** 2020-06-23

**Authors:** Xi-Min Qiao, Xiao-Feng Xu, Hao Zi, Guo-Xiong Liu, Bing-Hui Li, Xiang Du, Zhi-Hai Tian, Xiao-Ying Liu, Li-Sha Luo, Xiao Wang

**Affiliations:** ^1^Clinical Research Center of Xianyang City, Xianyang Central Hospital, Xianyang, China; ^2^Department of Urology, Xianyang Central Hospital, Xianyang, China; ^3^Center for Evidence-Based and Translational Medicine, Zhongnan Hospital of Wuhan University, Wuhan, China; ^4^Department of Neurology, Xianyang Central Hospital, Xianyang, China; ^5^Department of Radiology, Xianyang Central Hospital, Xianyang, China; ^6^Department of Infectious Disease, Xianyang Central Hospital, Xianyang, China; ^7^Center for Primary Health Care Research, Lund University/Region Skåne, Skåne University Hospital, Malmö, Sweden

**Keywords:** re-positive, COVID-19, SARS-CoV-2, discharge, follow-up

## Abstract

**Background:** The frequent emergence of the re-positive patients with COVID-19 is a potential threat worldwide. This study aimed to describe data from admission to follow-up for patients with COVID-19 and analyze the possible causes for re-positive nucleic acid tests to provide more scientific basis for reducing the numbers of re-positive patients after discharge.

**Methods:** We retrospectively recorded 15 patients with COVID-19 admitted to the Xianyang Central Hospital, China. The baseline, exposure histories, clinical syndromes, laboratory characteristics, nucleic acid, and follow-up tests were analyzed, and the radiological characteristics of re-positive patient at different periods were compared.

**Results:** Eight (53.33%) patients had the history of travel to Wuhan, four (26.67%) patients had close contact with confirmed patients, and one (6.67%) patient had close contact with suspected patients. After treatment, all patients had two consecutively negative nucleic acid tests and were discharged from hospital. All patients were followed up for more than 14 days, and the average time from discharge to the first follow-up was 14.67 ± 3.31 days (from 9 to 22 days). Most patients showed no clinical symptoms and negative nucleic acid tests, while one patient had an itchy throat, her CT scan showed a light density shadow in the right lower lobe of the lung, and the nucleic acid was once again positive. The second follow-up of the other 14 patients (except the re-positive one) was conducted 20.80 ± 7.78 days (from 13 to 30 days) after discharge, and all of them had negative nucleic acid tests. The positive patient was immediately readmitted and received a new round of treatment. Her family members and colleagues remained healthy until now.

**Conclusions:** The quality of nucleic acid testing reagents should be enhanced, and the training of nucleic acid sampling operators should be strengthened to reduce the false-negative results in the nucleic acid of SARS-CoV-2; the clinical specimens of throat and nasopharynx swabs can be collected at the same time; IgM- and IgG-specific antibodies of SARS-CoV-2 should be carried out for discharged patients; the radiological characteristics should be evaluated strictly; and the discharge standard can be specified according to the baseline and severity of disease of patients.

## Introduction

In December 2019, an unknown cause of pneumonia broke out in Wuhan, which was later defined by the WHO as coronavirus disease 2019 (COVID-19) (1–3). According to a report from the Chinese Center for Disease Control and Prevention, 80% of patients with COVID-19 were classified as having mild conditions in China (4). Most patients can be discharged from hospitals after a series of treatments, but they were required to maintain home quarantine for 2 weeks and return to hospital regularly for follow-up testing. Previous studies have shown that some patients exhibited positive nucleic tests after their discharge (5, 6). Xiao et al. reported 21.4% COVID-19 patients experienced a “turn positive” nucleic test after two consecutively negative results, and they thought the results may be caused by the false-negative of diagnosis test and prolonged nucleic acid conversion (7). Chen et al. reported one case of a COVID-19 patient who had a positive oropharyngeal swab test without clinical symptoms in her convalescence (8). Ye et al. reviewed 55 COVID-19 patients admitted in Zhongnan Hospital of Wuhan University, five of whom presented re-positive nucleic tests after discharge, and they concluded there may be no specific clinical symptoms to distinguish the re-positive patients who were discharged from hospitals (9). Loconsole et al. described a recurrent case with COVID-19 after recovery in Italy, and he developed new respiratory symptoms after the first follow-up visit (10). The frequent emergence of the re-positive cases indicated that there may be false-negative tests of detection kit or recurrence of the virus remaining in the body. Therefore, we should strictly evaluate the discharge patients to avoid any unnecessary transmission. To our knowledge, there was a lack of the systematic evidence on radical characteristics of discharged patients with re-positive nucleic tests, which was a crucial variable related to the clinical course and the appearance of the recurrence for patients with COVID-19. In the current study, we retrospectively recruited 15 discharged patients admitted in a designated hospital in Xianyang, Shaanxi province, China. The baseline information, exposure histories, clinical characteristics, laboratory tests, and nucleic acid tests at discharge, and follow-up from the 15 discharged patients were collected and analyzed, and the radiological characteristics of re-positive patient from admission, first discharge, follow-up visit, and second discharge were compared to provide more scientific evidence for larger cohort studies on re-positive patients with COVID-19 in the future.

## Methods

### Study Design

This was a retrospective study using data from patients with COVID-19 admitted to the Xianyang Central Hospital, which is a tertiary, comprehensive, teaching hospital and one that is designated for COVID-19 patients in Xianyang, Shaanxi province, China. A total of 17 patients were admitted in this hospital, two of whom were sent to a designated provincial hospital due to the severe/critical conditions. Hence, 15 patients were eventually enrolled in the study. This study was reviewed and approved by the Committee for Ethical Affairs of Xianyang Central Hospital, and we received informed consent from all 15 patients. The participants were confirmed based on the diagnostic criteria of the National Health Committee of the People's Republic of China and a real-time RT-PCR was used to detect positive nucleic acid of SARS-CoV-2 (11, 12). Discharged patients were to meet the following conditions: body temperature remained normal for more than 3 days; respiratory symptoms improved obviously; chest X-ray or CT showed significant improvement of exudative lesions; two consecutive respiratory samples that tested negative for nucleic acid, and the sample collection time was at least 24 h apart. After discharge, all patients underwent 14 days isolation and health surveillance at home. During the follow-up period, nucleic acid detection of respiratory tract samples was performed twice, and the time points were the 2nd and 4th weeks after discharge (3). Respiratory samples (throat swabs) were tested for nucleic acids at the Xianyang Center for Disease Control and Prevention.

### Data Collection

Hospitalization data were obtained from medical records through a customized data collection form. Follow-up data were obtained by direct contact with patients and outpatients review. We extracted the demographic data, exposure histories, clinical syndromes, laboratory characteristics, chest CT scans, and nucleic acid tests for all 15 confirmed patients.

### Statistical Analysis

Categorical variables were described as counts and percentages, and continuous variables were showed as a Mean ± standard deviation (*SD*). The data analysis was performed by the SAS software, version 9.4 TS1M6 (SAS Institute Inc, Cary, NC), and it was visualized by Microsoft PowerPoint 2016.

## Results

**Figure 1:**
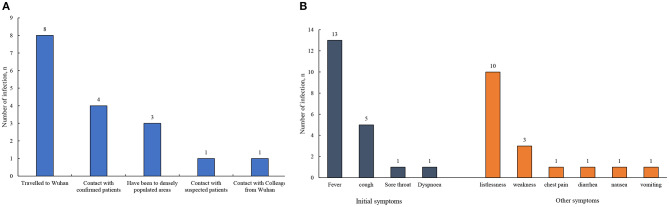
**(A,B)** The exposure histories and clinical symptoms of the patients with COVID-19.

In Table 1, we presented the clinical and demographic characteristics of all 15 COVID-19 patients (P1–P15). Of them, eight were male and seven were female, and the age ranged from 9 to 62 years. For the exposure history, there were eight (53.33%) patients with the history of traveling to Wuhan, four (26.67%) patients with close contact with confirmed patients, one (6.67%) patient with close contact with suspected patients, and one (6.67%) patient with close contact with colleague from Wuhan (Figure 1A). A total of 13 (86.67%) patients were hospitalized with fever as the initial symptom, and five patients were accompanied by cough. Other general symptoms such as listlessness, weakness, and diarrhea were also observed (Figure 1B). Of the patients, 93.33% exhibited abnormal CT scans during hospitalization. Most of the patients were confirmed positive at least once by the nucleic acid tests. The length of stay for all patients was 17 ± 3.80 days. After treatment, all patients had normal white blood cell and neutrophil counts, while one patient's lymphocyte count bellowed the normal range. Additionally, the nucleic acid of all patients had changed to negative, while 80% still had abnormal CT scans at discharge from the hospital (Table 1).

**Table 1:** The exposure history and characteristics of COVID-19 patients during hospitalization and at discharge.

**Variables**	**P1**	**P2**	**P3**	**P4**	**P5**	**P6**	**P7**	**P8**	**P9**	**P10**	**P11**	**P12**	**P13**	**P14**	**P15**	***N* (%) / Mean ±*SD***
Age	23	42	26	26	30	22	9	40	62	32	41	59	41	41	57	36.73 ± 14.91
Sex	Female	Male	Male	Male	Female	Female	Male	Male	Female	Male	Female	Female	Male	Female	Male	8 (53.33%)
**Exposure history**																
Contact with confirmed patients	No	No	No	No	No	No	Yes	Yes	No	No	No	Yes	Yes	No	No	4 (26.67%)
Contact with suspected patients	No	No	No	No	No	No	No	No	No	No	No	No	No	No	Yes	1 (6.67%)
Contact with wild animals	No	No	No	No	No	No	No	No	No	No	No	No	No	No	No	0 (0%)
Traveled to Wuhan	Yes	Yes	Yes	Yes	No	No	No	Yes	Yes	No	No	No	Yes	Yes	No	8 (53.33%)
**Characteristics during hospitalization**																
Initial symptoms	Fever	Fever	Sore throat	Fever	Dyspnoea	Fever	Fever	Fever	Fever	Fever	Fever	Fever	Fever	Fever	Fever	-
								Cough	Cough		Cough		Cough		Cough	
Other symptoms	Chest pain	Weakness	Listlessness	Listlessness	Listlessness	Listlessness	Listlessness	Listlessness	Nausea	Cough	Listlessness	Weakness	Listlessness	Weakness	Listlessness	-
								Diarrhea	Vomiting	Listlessness						
The highest temperature (°C)	37.5	37.8	37	37.5	36.8	37.6	37.5	38.5	37.5	37.4	38.3	38	38.4	39	38	37.79 ± 0.59
Interval between symptom onset and diagnosis (days)	8	4	3	5	0	2	11	11	8	7	9	3	4	3	7	5.67 ± 3.33
CT	Positive	Positive	Positive	Positive	Positive	Positive	Negative	Positive	Positive	Positive	Positive	Positive	Positive	Positive	Positive	14 (93.33%)
Test times of nucleic acid positive	2	2	1	1	1	2	2	2	1	1	1	1	1	1	1	-
Length of stay (days)	10	19	13	14	14	17	15	17	21	17	18	18	22	25	15	17.00 ± 3.80
**Characteristics at discharge**																
White blood cell (*10^9^/L)	5.05	7.69	5.18	4.53	6.99	6.88	5.1	3.91	5.81	4.54	4.45	5.94	5.13	8.93	4.38	5.63 ± 1.42
Lymphocyte (*10^9^/L)	1.55	2.98	1.52	1.83	2.68	1.92	2.71	1.16	1.34	1.48	0.97	1.91	1.56	2.14	1.14	1.79 ± 0.61
Neutrophil (*10^9^/L)	3.02	3.81	3.13	2.12	3.58	4.44	1.6	2.21	4.1	2.65	3.31	2.86	3.03	6.05	2.82	3.25 ± 1.08
PCT (ng/mL)	<0.1	0.11	<0.1	<0.1	0.13	<0.1	0.36	0.18	0.11	0.61	<0.1	0.29	0.13	0.16	0.33	-
CT	Negative	Positive	Positive	Positive	Positive	Positive	Negative	Positive	Positive	Positive	Positive	Positive	Negative	Positive	Positive	12 (80%)
Nucleic acid test	Negative	Negative	Negative	Negative	Negative	Negative	Negative	Negative	Negative	Negative	Negative	Negative	Negative	Negative	Negative	0 (0%)

All the patients were followed-up for more than 14 days, and their clinical and laboratory follow-up results were shown in Table 2. The average time from discharge to the first follow-up visit was 14.67 ± 3.31 days (from 9 to 22 days), and no patients had clinical symptoms at the first follow-up. Among the 15 infected patients, two cases had higher neutrophil counts (8.02 × 10^9^/L), while none of the patients had white cell count below the normal range. Although there were still 11 patients with abnormal CT scans, the lesions have been absorbed and improved compared with those at the time of discharge. It was remarkable that the nucleic acid test of P5 turned positive again, and she was immediately readmitted to the hospital.

**Table 2:** The follow-up results of discharged patients with COVID-19.

**Variables**	**P1**	**P2**	**P3**	**P4**	**P5^**†**^**	**P6**	**P7**	**P8**	**P9**	**P10**	**P11**	**P12**	**P13**	**P14**	**P15**	***N* (%) / Mean ±*SD***
**The first follow-Up**																
Interval between discharge and follow-up (days)	22	20	17	15	15	16	12	10	9	14	14	14	14	14	14	14.67 ± 3.31
Clinical symptoms	No	No	No	No	Itchy throat	No	No	No	No	No	No	No	No	No	No	0 (0%)
Temperature (°C)	Normal	Normal	Normal	Normal	Normal	Normal	Normal	Normal	Normal	Normal	Normal	Normal	Normal	Normal	Normal	0 (0%)
White blood cell (*10^9^/L)	7.13	7.4	4.39	5.43	5.01	6.75	7.4	4.46	5.9	6.8	5.94	5.89	10.56	5.94	4.98	6.27 ± 1.55
Lymphocyte (*10^9^/L)	1.51	2.45	1.06	2.74	2.14	1.91	4.29	1.5	1.8	1.71	1.18	1.92	1.78	1.18	1.27	1.90 ± 0.81
Neutrophil	5.18	4.15	2.85	1.94	2.25	4.32	2.12	2.23	3.51	4.24	4.32	3.56	8.02	8.02	3.22	4.00 ± 1.90
Lymphocyte percentage (%)	21.2	33.1	24.1	50.46	42.7	28.3	58	33.6	30.5	25.1	19.9	32.6	16.9	19.9	25.5	30.79 ± 11.71
CT	Negative	Positive	Negative	Positive	Positive	Positive	Negative	Positive	Positive	Positive	Positive	Positive	Negative	Positive	Positive	11 (73.33%)
Nucleic acid test	Negative	Negative	Negative	Negative	Positive	Negative	Negative	Negative	Negative	Negative	Negative	Negative	Negative	Negative	Negative	1 (6.67%)
**The second follow-up**					**The second discharge**											
Interval between discharge and follow-up (days)	30	28	28	28	**-**	28	17	13	14	21	21	21	21	21	21	20.80 ± 7.78
Clinical symptoms	No	No	No	No	**No**	No	No	No	No	No	No	No	No+	No	No	0 (0%)
Temperature (°C)	Normal	Normal	Normal	Normal	**Normal**	Normal	Normal	Normal	Normal	Normal	Normal	Normal	Normal	Normal	Normal	0 (0%)
White blood cell (*10^9^/L)	6.95	7.70	5.68	5.75	**6.75**	4.27	7.40	4.46	5.37	8.06	5.62	5.50	6.63	7.59	6.21	5.81 ± 1.98
Lymphocyte (*10^9^/L)	1.43	2.93	1.45	2.95	**2.30**	1.98	4.29	1.50	1.78	1.96	0.88	1.82	1.97	2.04	1.03	1.86 ± 1.00
Neutrophil (*10^9^/L)	5.02	4.20	3.72	2.07	**3.65**	1.94	1.92	2.77	3.11	5.28	4.48	3.40	3.98	4.90	4.75	3.44 ± 1.48
Lymphocyte percentage (%)	20.60	38.10	25.50	51.30	**34.1**	46.40	58.00	33.60	33.10	24.30	15.70	33.10	29.70	26.90	16.60	30.19 ± 14.73
CT	Negative	Positive	Negative	Negative	**Negative**	Positive	Negative	Positive	Positive	Positive	Positive	Negative	Negative	Negative	Positive	7 (50%)
Nucleic acid test	Negative	Negative	Negative	Negative	**Negative**	Negative	Negative	Negative	Negative	Negative	Negative	Negative	Negative	Negative	Negative	0 (0%)

† *P5 were directly hospitalized after the first follow-up, so the second follow-up result of P5 is the second discharge result on March 17, 2020*.

The second follow-up of the other 14 patients (except P5) was conducted 20.80 ± 7.78 days (from 13 to 30 days) after discharge, and none of the patients had clinical symptoms. The laboratory results showed that none of patients had white cell count below the normal range, while two cases had lower lymphocyte counts. The CT scans of all 14 patients for the second follow-up were significantly improved compared with those for the first follow-up, and all patients had negative nucleic acid tests (Table 2).

**Figure 2:**
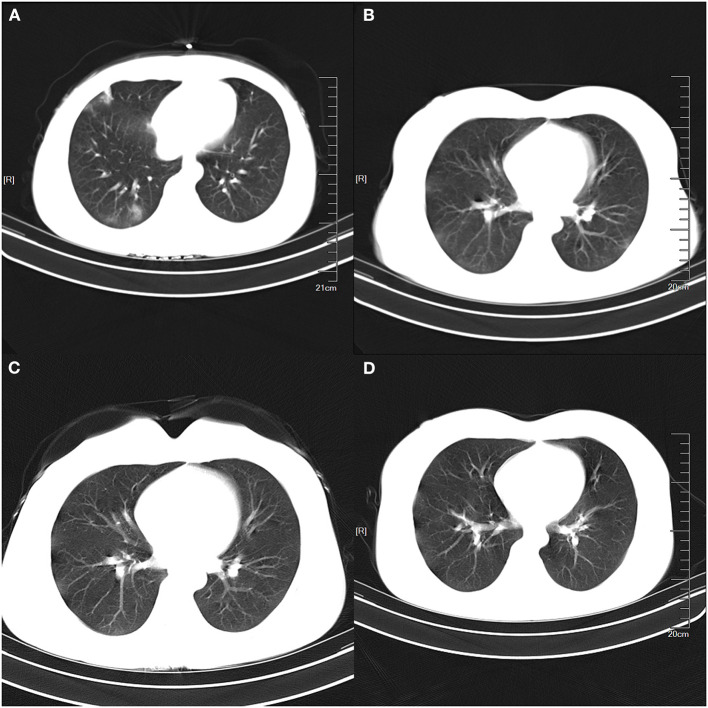
The specific Chest CT scans of the re-positive patient at different periods. **(A)** was the multiple patchy high-density shadow on bilateral lungs at admission; **(B)** was the patchy low-density shadows at first discharge, which was significantly better than that of admission; **(C)** showed patchy low-density shadows in the right lower lobe at the first follow-up visit; and **(D)** showed no abnormal high-density shadows at second discharge.

P5 was the only patient with re-positive nucleic acid test in follow-up visit after discharge. She had close contact with her colleague who was from Wuhan on 20 January, 2020 and then isolated herself at home immediately when she knew the travel history of her colleague; she did not have any contact with family members during her isolation period. After 10 days of isolation, she felt panicky and proactively asked to perform the relevant examination. The CT scan indicated multiple patchy high-density shadows on bilateral lungs. The nucleic acid result detected by the local CDC was positive, and she was diagnosed with COVID-19 and admitted to the hospital on 31 January 2020. She underwent a series of treatment during 14-days stay in hospital; her symptoms disappeared, two consecutive nucleic acid tests were negative, and the CT image showed that the infectious lesions in both lungs was significantly better than those at admission. The patient was allowed to leave the hospital and be isolated at home again. She returned to the hospital for the first follow-up 15 days after discharge according to the government's guideline. The patient felt itchy throat, occasional discomfort in the right chest, occasional coughing, and expectoration, while the temperature was normal. The laboratory tests showed that the white blood cell was 5.01 × 10^9^/L, lymphocyte was 2.14 × 10^9^/L, neutrophil was 2.25 × 10^9^/L and lymphocyte percentage was 42.7%. Additionally, the CT scan showed the light density shadow in the right lower lobe of the lung, which was better than that of discharge. Unfortunately, her nucleic acid was returned as positive in this follow-up test (Table 2), and she was thus immediately readmitted to the hospital and received a new round of treatment. She was discharged again on March 17, 2020 without any clinical symptom, and temperature (°C) was normal, white blood cell was 6.75 × 10^9^/L, lymphocyte was 2.30 × 10^9^/L, neutrophil was 3.65 × 10^9^/L, and lymphocyte percentage was 34.1%. The nucleic acid test was negative and CT scan showed no abnormal shadow in both lungs. The specific chest CT scans at different periods were shown in Figure 2. Remarkably, her colleague who had traveled to Wuhan didn't have any symptoms until now and her family members were all healthy.

## Discussion

COVID-19 is the third coronavirus epidemic in the twenty-first century after severe acute respiratory syndrome (SARS) and Middle East respiratory syndrome (MERS) (13, 14). SARS-CoV-2 is highly infectious and continues to spread worldwide. It has been reported that there were more than 100,000 infected cases with COVID-19 and more than 3,500 deaths globally (15). Two consecutively negative nucleic acid tests are the most important discharge criteria for COVID-19 patients. However, it has been reported that a few patients have positive nucleic acid again after discharge (12).

In our study, one case among 15 infected patients had the re-positive nucleic acid result after discharge. She had no contact with the confirmed or suspected patient and Wuhan travel history but had close contact with a colleague who was from Wuhan on 20 January 2020. The patient began to show symptoms after 10 days of isolation at home, and she was admitted to hospital with panicky as the initial symptom. After 14 days of hospitalization, all clinical symptoms disappeared and the CT result was also significantly better than that at the time of admission, meeting the Chinese discharge criteria for COVID-19 patients. However, she developed symptoms again when she returned to the hospital for reexamination for the first time, and the CT scan showed there was still a light density shadow in the right lung. At the same time, her nucleic acid test turned positive again, which can be interpreted by some possible reasons. Firstly, the patient improved significantly after a period of drug treatment, but not completely cured. The nucleic acid test at the time of discharge may appear false-negative, and as we know, the poorer quality and longer storage time of the samples might be one of the reasons that patients showed false-negative test. In our study, throat swab was collected and analyzed when the patient was discharged, and the re-positive test may be caused by the irregular operation and storage of sample, and poor nucleic acid detection reagents. Secondly, the virus was recurred after the patient's discharge. The previous study reported that the patients' immune function also plays an important role in the recovery, while the SARS-CoV-2 will be occasionally positive when it is not completely cleared in the body. Although more than 10 patients with positive nucleic acid were followed by experts in Hong Kong, no live virus was cultured in P3 laboratory, which suggests that the sample in nucleic acid detection may be the nucleic acid fragment of the SARS-CoV-2. However, the re-positive test in our study may be not a part of the virus genome that still remained in the throat because the patient was accompanied by obvious clinical symptoms and the lesions in the lung. Additionally, the patient had been isolated at home since she was discharged from the hospital and had not been exposed to other confirmed or suspected patients, which indicated her re-positive test may be not the result of re-infection, and from the perspective of etiology, the recovered patients have stronger resistance to the SARS-CoV-2 because of their antibodies. Therefore, the chance of repeated infection is very small for most of the patients.

Although there is no study proving that re-positive patients can transmit SARS-CoV-2 to others, it still needs to be paid attention to the management of discharged patients. In order to reduce the false-negative results in the nucleic acid test of SARS-CoV-2, the quality of nucleic acid testing reagents should be enhanced, and the training of nucleic acid sampling operators should be strengthened to ensure that the sampling process is standardized and the operation is accurate. Additionally, the hospital should advocate to collect clinical specimens of throat and nasopharynx swabs at the same time. In order to ensure the patients are completely cured, IgM- and IgG-specific antibody of SARS-CoV-2 should be carried out for all discharged patients. Additionally, hospitals can specify the discharge standard according to the baseline, severity of disease, and other factors of patients. The seventh edition guideline for the diagnosis and treatment of COVID-19, issued by the National Health Committee of the People's Republic of China, indicated that the discharged patients with COVID-19 should be isolated in a designated hospital or at home, have their health status monitored for 14 days, and have a reexamination performed 2 and 4 weeks after discharge to ensure the recovery of patients and protect health population around the recovered patients from infection (16, 17). In addition, our study indicated that the CT scan of re-positive patient still showed abnormal lesions in the right lung at the first follow-up visit, so more rigorous criteria should be evaluated for the result of radiology to reduce the possibility of the re-positive nucleic acid.

The main limitation of this study is that it was a single-center retrospective study with a small sample size; although we included all admitted patients in the hospital, there would be unavoidable inherent bias upon collection. A multi-center prospective study with larger samples needs to be conducted to further verify the conclusions in the present study. Additionally, our study did not provide the IgG- and IgM-specific antibodies of patients due to unavailability of the data.

In conclusion, the re-positive nucleic acid tests for COVID-19 patients may be caused by false-negative tests, prolonged storage of samples, and recurrence of virus remaining in the body. Therefore, the quality of nucleic acid testing reagents should be enhanced, and the training of nucleic acid sampling operators should be strengthened to reduce the false-negative results in the nucleic acid test of SARS-CoV-2; the clinical specimens of throat and nasopharynx swabs can be collected at the same time; and IgM- and IgG-specific antibodies of SARS-CoV-2 should be carried out for all discharged patients. Additionally, the radiological characteristics should be evaluated strictly, and the discharge standard can be specified according to the baseline, severity of disease, and other factors of patients.

## Data Availability Statement

All datasets presented in this study are included in the article/supplementary material.

## Ethics Statement

The studies involving human participants were reviewed and approved by Committee for Ethical Affairs of Xianyang Central Hospital. Written informed consent to participate in this study was provided by the participants' legal guardian/next of kin. Written informed consent was obtained from the individual(s) for the publication of any potentially identifiable images or data included in this article.

## Author Contributions

X-MQ, L-SL, and XW: conception and design. XD, Z-HT, G-XL, and X-FX: collection and assembly of data. HZ, X-FX, B-HL, and G-XL: analysis and interpretation of the data. B-HL, X-YL, and L-SL: statistical expertise. L-SL, X-FX, and HZ: drafting of the manuscript. Z-HT, XD, X-MQ, XW, and L-SL: critical revision of the article for important intellectual content. All authors had full access to all of the data in the study and take responsibility for the integrity of the data and the accuracy of the data analysis.

## Conflict of Interest

The authors declare that the research was conducted in the absence of any commercial or financial relationships that could be construed as a potential conflict of interest.
